# GraTeLPy: graph-theoretic linear stability analysis

**DOI:** 10.1186/1752-0509-8-22

**Published:** 2014-02-27

**Authors:** Georg R Walther, Matthew Hartley, Maya Mincheva

**Affiliations:** 1 Computational and Systems Biology, John Innes Centre, Norwich Research Park, Norwich, UK; 2 Department of Mathematical Sciences, Northern Illinois University, DeKalb, IL 60115, USA

**Keywords:** Biochemical mechanism, Bipartite digraph, Multistability, Turing instability, Oscillations, Parameter-free model discrimination

## Abstract

**Background:**

A biochemical mechanism with mass action kinetics can be represented as a directed bipartite graph (bipartite digraph), and modeled by a system of differential equations. If the differential equations (DE) model can give rise to some instability such as multistability or Turing instability, then the bipartite digraph contains a structure referred to as a critical fragment. In some cases the existence of a critical fragment indicates that the DE model can display oscillations for some parameter values. We have implemented a graph-theoretic method that identifies the critical fragments of the bipartite digraph of a biochemical mechanism.

**Results:**

GraTeLPy lists all critical fragments of the bipartite digraph of a given biochemical mechanism, thus enabling a preliminary analysis on the potential of a biochemical mechanism for some instability based on its topological structure. The correctness of the implementation is supported by multiple examples. The code is implemented in Python, relies on open software, and is available under the GNU General Public License.

**Conclusions:**

GraTeLPy can be used by researchers to test large biochemical mechanisms with mass action kinetics for their capacity for multistability, oscillations and Turing instability.

## Background

Biochemical mechanisms are often modeled by differential equations (DE) systems. Instabilities, such as multistability, oscillations, or Turing instability, are ubiquitous in DE models of biochemical mechanisms. Methods from bifurcation analysis are usually applied in order to analyze DE models for instabilities [1]. Bifurcation analysis methods are easily applied when the DE model has one or two concentration species (phase plane analysis) or has a relatively small number of parameters (numerical bifurcation analysis). However, it is both difficult and expensive to apply bifurcation methods to analyze large DE models with many variables for instabilities.

On the other hand a biochemical mechanism can be represented as a directed bipartite graph (bipartite digraph), which is a graph with two different sets of nodes representing species and reactions, and directed edges connecting a species and a reaction node. The existence of structures referred to as critical fragments in the bipartite digraph of a biochemical mechanism is necessary for the existence of multistability or Turing instability in the DE model [2-4]. Thus biochemical mechanisms that do not have the potential for multistability or Turing instability can be ruled out early in the modeling process. The existence of a critical fragment that does not contain all species nodes can indicate that oscillations exist for some parameter values for the DE model [3]. Thus graph-theoretic methods can be used to determine the potential of various biochemical mechanisms to exhibit some desired behavior, including multistability related to cell decision [5,6], oscillations related to circadian rhythms [7], or Turing instability related to pattern formation [8].

Graph-theoretic methods are applicable to mechanisms with any number of species and reactions, which enables the screening of large biochemical mechanisms for potential instabilities. However, application of graph-theoretic methods by hand becomes challenging for large mechanisms, making a computational implementation highly desirable.

The graph-theoretic method implemented by GraTeLPy identifies all critical fragments in the bipartite digraph of a biochemical mechanism that can give rise to some instability (multistability, oscillations and Turing instability) [3,4]. GraTelPy is implemented in Python and can run in parallel on computer clusters which increases the size of testable biochemical mechanisms.

Other software packages implement theoretical and computational methods for studying chemical reaction networks for multistability. Using the deficiency theory developed by M. Feinberg and collaborators [9], it can be shown that a chemical network model does not admit multistability for any choice of parameter values. The CRNT toolbox [10] developed originally by M. Feinberg implements the Deficiency One algorithm [11], that can be used to detect if a given network has the capacity for multistability [12]. If a given network admits multiple positive equilibria, in many cases the CRNT toolbox returns rate constant values such that the corresponding model system has at least two positive equilibria. In recent years, the CRNT toolbox has been extended to implement an algorithm for the mass-action injectivity test. A special case of this test is the Jacobian criterion, which provides a sufficient condition for excluding the existence of multiple positive equilibria and is based on the theory developed in [2,13,14].

Related software packages include BioNetX [15] and CoNtRol [16]. BioNetX is based on the work of M. Banaji and G. Craciun [17,18] and is created by C. Pantea. BioNetX is used to analyze uni-molecular and bi-molecular reaction networks for the existence of multiple positive equilibria in [19,20]. CoNtRol [16] is a web-based software package that employs matrix and graph-theoretic methods based on the DSR graph [17,18,21]. In particular CoNtRol provides information about the capacity of a given chemical network for multistability based on the DSR graph and on some additional tests. In addition CoNtRol calculates the deficiency of a network and checks if a network is weakly reversible. BioNetX and CoNtRol are available to download for free, they are open-source and are conveniently web-based.

We describe in Section Mathematical background the DE model and the bipartite digraph of a biochemical mechanism, as well as the instability criteria. In Section Implementation we describe the algorithm for finding critical fragments. In Section Results and discussion we present several examples along with some concluding remarks. A guide for downloading and installing GraTeLPy for Mac, Windows and Linux operating systems is available in the Additional file 1.

## Mathematical background

Here we introduce the differential equations model and the bipartite digraph representation of a biochemical mechanism. In this section we also briefly describe the instability criteria for multistability, oscillations and Turing instability. More details on the instability criteria are available in [3,4].

### Mathematical model

A biochemical mechanism with*n* species *A*_*i*_, *i*=1,…,*n*, and *m* elementary reactions *B*_*j*_ can be written as

(1) $$B_{j}:\;\overset{n}{\underset{i=1}{\sum }}\alpha_{\text{ji}}A_{i}\overset{k_{j}}{\underset{}{\to }}\overset{n}{\underset{i=1}{\sum }}\beta_{\text{ji}}A_{i},\;j=1,\ldots ,m,$$

where *k*_*j*_>0, *j*=1,…,*m* are the *rate constants*. The constants *α*_*ji*_≥0 and *β*_*ji*_≥0 in (1) are small integers called *stoichiometric coefficients* that account for the number of molecules of species *A*_*i*_ participating in the *j*^th^ elementary reaction. An example of a biochemical mechanism, the reversible substrate inhibition mechanism, is given below:

(2) $$\begin{array}{ll} B_{1}: & \;A_{1}\overset{k_{1}}{\underset{}{\to }}\emptyset ,\\ B_{2}: & \;\emptyset \overset{k_{2}}{\underset{}{\to }}A_{1},\\ B_{3}: & \;A_{1}+A_{2}\overset{k_{3}}{\underset{}{\to }}A_{3},\\ B_{4}: & \;A_{3}\overset{k_{4}}{\underset{}{\to }}A_{2},\\ B_{5}: & \;A_{1}+A_{3}\overset{k_{5}}{\underset{}{\to }}A_{4},\\ B_{6}: & \;A_{4}\overset{k_{6}}{\underset{}{\to }}A_{1}+A_{3}, \end{array}$$

where the first two reactions represent an inflow and outflow reaction, respectively.

We will assume that every species *A*_*k*_ in (1) is consumed and produced in at least one true reaction, i.e, a reaction which is different from an outflow reaction *A*_*k*_→*∅* or an inflow reaction *∅*→*A*_*k*_. However, we do not specifically require that all species participate in an inflow and an outflow reaction.

We further assume *mass action kinetics* for the mechanism (1) with *rate functions*

(3) $$w_{j}=k_{j}u_{1}^{\alpha_{j1}}\ldots u_{n}^{\alpha_{\text{jn}}},\;\;j=1,\ldots ,m,$$

where *u*_*k*_(*t*) is the concentration at time *t* of a species *A*_*k*_, *k*=1,…,*n*.

The ordinary differential equations (ODE) model of a mass-action biochemical mechanism (1) can be written in vector form as

(4) $$\dot{u}(t)=\text{Sw}(u),$$

where *u*(*t*)=(*u*_1_(*t*),…,*u*_*n*_(*t*))^*T*^ is the concentration vector of the chemical species of (1), *S*_*ji*_=*β*_*ji*_−*α*_*ji*_ are the entries of the stoichiometric matrix *S* and *w*(*u*)=(*w*_1_(*u*),…,*w*_*m*_(*u*))^*T*^ is the vector of rate functions (3). *Throughout the paper it will be assumed that the ODE system (*4*) has a positive equilibrium.*

The model equations of the reversible substrate mechanism (2) are given below

(5) $$\begin{array}{ll} {\dot{u}}_{1} & =-k_{1}u_{1}+k_{2}-k_{3}u_{1}u_{2}-k_{5}u_{1}u_{3}+k_{6}u_{4},\\ {\dot{u}}_{2} & =-k_{3}u_{1}u_{2}+k_{4}u_{3},\\ {\dot{u}}_{3} & =k_{3}u_{1}u_{2}-k_{4}u_{3}-k_{5}u_{1}u_{3}+k_{6}u_{4},\\ {\dot{u}}_{4} & =k_{5}u_{1}u_{3}-k_{6}u_{4}. \end{array}$$

The rank of the stoichiometric matrix *S* of (5) equals 3 since there is one conservation relationship *u*_2_+*u*_3_+*u*_4_=*c*_0_.

Since

$${\dot{u}}_{i}(t)=f_{i}(u)=\overset{m}{\underset{j=1}{\sum }}S_{\text{ji}}w_{j}(u)$$

where *S*_*ji*_ are the stoichiometric matrix entries and *w*_*j*_(*u*) are the rate functions (3), the Jacobian matrix *J*(*u*,*w*) has entries that can be written as

(6) $$J_{\text{ik}}(u,w)=\frac{\partial f_{i}}{\partial u_{k}}=\overset{m}{\underset{j=1}{\sum }}S_{\text{ji}}\alpha_{\text{jk}}\frac{w_{j}}{u_{k}}.$$

Note that the concentrations *u*_*k*_, *k*=1,…,*n* and the rate functions *w*_*j*_(*u*), *j*=1,…,*m* (both considered evaluated at a positive equilibrium) are used as parameters in (6). The rank of the Jacobian (6) equals the rank of the stoichiometric matrix *S*[3].

The Jacobian matrix of the model (5) parametrized in (*u*,*w*) has rank 3 and is given below

(7) $$J(u,w)=\left(\begin{array}{cccc} -\frac{w_{1}+w_{3}+w_{5}}{u_{1}} & -\frac{w_{3}}{u_{2}} & -\frac{w_{5}}{u_{3}} & \frac{w_{6}}{u_{4}}\\ -\frac{w_{3}}{u_{1}} & -\frac{w_{3}}{u_{2}} & \frac{w_{4}}{u_{3}} & 0\\ \frac{w_{3}-w_{5}}{u_{1}} & \frac{w_{3}}{u_{2}} & -\frac{w_{3}+w_{4}}{u_{3}} & -\frac{w_{6}}{u_{4}}\\ \frac{w_{5}}{u_{1}} & 0 & \frac{w_{5}}{u_{3}} & -\frac{w_{6}}{u_{4}} \end{array}\right)\;.$$

The characteristic polynomial of *J*(*u*,*w*) is

(8) $$P(\lambda )=\det(\;J(u,w)-\mathrm{\lambda I})=\overset{n}{\underset{k=0}{\sum }}a_{k}(u,w)\lambda^{n-k}\;,$$

where *I* is the identity matrix. Note that the coefficients *a*_*i*_=*a*_*i*_(*u*,*w*), *i*=1,…,*n* of (8) are also functions of (*u*,*w*). For example, the last non-zero coefficient of the characteristic polynomial of the Jacobian (7) is

(9) $$\;a_{3}(u,w)=\frac{w_{4}w_{6}(w_{1}+w_{3})}{u_{1}u_{3}u_{4}}+\frac{w_{1}w_{3}w_{6}}{u_{1}u_{2}u_{4}}+\frac{w_{3}w_{5}(w_{1}-w_{4})}{u_{1}u_{2}u_{3}}.$$

### The bipartite digraph of a biochemical mechanism

For the convenience of the reader, in this section we present definitions regarding the bipartite digraph of a biochemical mechanism (1) [3,4,22]. To illustrate the definitions in this section we will continue to use as an example the reversible substrate mechanism (2).

A *directed bipartite graph* (bipartite digraph) has a node set that consists of two disjoint subsets, *V*_1_ and *V*_2_, and each of its directed edges (arcs) has one end in *V*_1_ and the other in *V*_2_[23].

**Figure 1:**
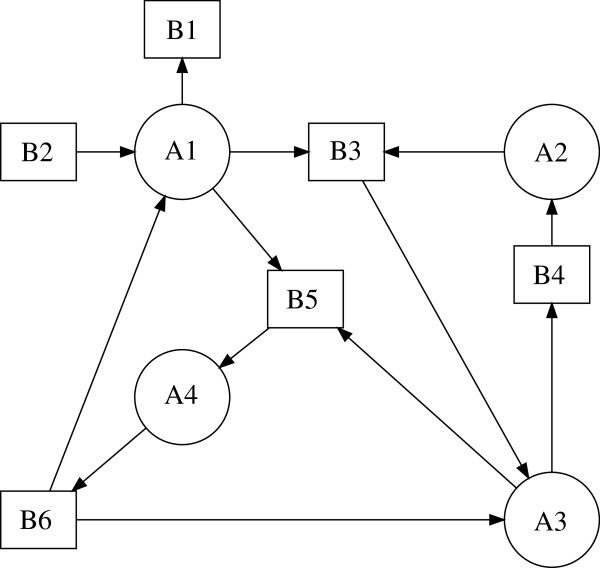
**Bipartite digraph of the reversible substrate inhibition mechanism.** Bipartite digraph of the reversible reaction mechanism (2). Circles denote species nodes and squares denote reaction nodes of the mechanism.

The *bipartite digraphG* of a biochemical reaction network (1) is defined as follows. The nodes are separated into two sets, one for the chemical species *V*_1_={*A*_1_,*A*_2_,…,*A*_*n*_} and one for the elementary reactions *V*_2_={*B*_1_,*B*_2_,…,*B*_*m*_}. We draw an arc from *A*_*k*_ to *B*_*j*_ if and only if species *A*_*k*_ is a reactant in reaction *j*, i.e., if the stoichiometric coefficient *α*_*jk*_>0 in (1). Similarly, we draw an arc from *B*_*j*_ to *A*_*i*_ if and only if *A*_*i*_ is a product in reaction *j*, i.e., if the stoichiometric coefficient *β*_*ji*_>0 in (1). Therefore the set of arcs *E*(*G*) consists of arcs such as (*A*_*k*_,*B*_*j*_) and (*B*_*j*_,*A*_*i*_). Hence the bipartite digraph can be defined as *G*={*V*,*E*(*G*)} where *V*=*V*_1_∪*V*_2_ is the set of nodes and *E*(*G*) is the set of arcs. If an arc is not weighted explicitly, we assume that its weight equals 1. The corresponding bipartite digraph of the reversible substrate inhibition mechanism (2) is shown in Figure 1.

The element [ *A*_*k*_,*B*_*j*_] is an *edge* if *α*_*jk*_>0, i.e., if species *A*_*k*_ is a reactant in reaction *j*. The *weight of an edgeE*=[ *A*_*k*_,*B*_*j*_] is defined as

(10) $$K_{E}=-\alpha_{\text{jk}}^{2}.$$

For example, the edge *E*=[ *A*_1_,*B*_3_] in Figure 1 has weight *K*_*E*_=−1.

If *α*_*jk*_*β*_*ji*_>0, then the arcs (*A*_*k*_,*B*_*j*_) and (*B*_*j*_,*A*_*i*_) form a *positive path* [ *A*_*k*_,*B*_*j*_,*A*_*i*_] that corresponds to the production of *A*_*i*_ from *A*_*k*_ in a reaction *j*. The weight of the positive path [ *A*_*k*_,*B*_*j*_,*A*_*i*_] is defined as *α*_*jk*_*β*_*ji*_. For example, the positive path [ *A*_1_,*B*_3_,*A*_3_] in Figure 1 has weight 1.

If *α*_*jk*_*α*_*ji*_>0, then the arcs (*A*_*k*_,*B*_*j*_) and (*A*_*i*_,*B*_*j*_) form a *negative path*$\left[\;\bar{A_{k},B_{j},A_{i}}\right]$ that corresponds to *A*_*k*_ and *A*_*i*_ interacting as reactants in reaction *j*. The weight of the negative path $\left[\;\bar{A_{k},B_{j},A_{i}}\right]$ is defined as −*α*_*jk*_*α*_*ji*_. Note that the negative paths $\left[\;\bar{A_{k},B_{j},A_{i}}\right]$ and $\left[\;\bar{A_{i},B_{j},A_{k}}\right]$ are considered to be different since they start at a different species node. For example, both $\left[\;\bar{A_{1},B_{3},A_{2}}\right]$ and $\left[\;\bar{A_{2},B_{3},A_{1}}\right]$ in Figure 1 are negative paths with weight −1. We note that the direction of the arcs is followed in the positive paths but not in the negative paths.

A *cycleC* of *G* is a sequence of distinct paths with the last species node of each path being the same as the first species node of the next path $C=\{(A_{i_{1}},B_{j_{1}},A_{i_{2}})$, $\left(A_{i_{2}},B_{j_{2}},A_{i_{3}}\right)$, …, $\left(A_{i_{k-1}},B_{j_{k-1}},A_{i_{k}}\right)$, $\left(A_{i_{k}},B_{j_{k}},A_{i_{1}})\right\}$. A cycle will be denoted by C=Ai1,Ai2,…,AikBj1,Bj2,…,Bjk, where the number of species nodes defines its *order*. The set of species nodes in a cycle is distinct, but there may be a repetition among the reaction nodes. This is because negative paths containing the same nodes are considered different depending on the starting species node. For example, C=A1,A2B3,B3 in Figure 1 is a cycle formed by the two negative paths $\left[\;\bar{A_{1},B_{3},A_{2}}\right]$ and $\left[\;\bar{A_{2},B_{3},A_{1}}\right]$.

A cycle is *positive* if it contains an even number of negative paths and *negative* if it contains an odd number of negative paths. The sign of a cycle *C* can also be determined by the *cycle weight* which is a product of all corresponding weights of negative and positive paths of *C*

(11) $$K_{C}=\underset{\bar{\left[A_{k},B_{j},A_{i}\right]}\in C}{\prod }(-\alpha_{\text{jk}}\alpha_{\text{ji}})\underset{[A_{k},B_{j},A_{i}]\in C}{\prod }\alpha_{\text{jk}}\beta_{\text{ji}}.$$

For example, C=A1,A3B3,B5 (see Figure 1) is a negative cycle of order 2 with weight *K*_*C*_=−1. The cycle C=A2,A3B3,B4 (see Figure 1) is a positive cycle of order 2 with weight *K*_*C*_=1.

**Figure 2:**
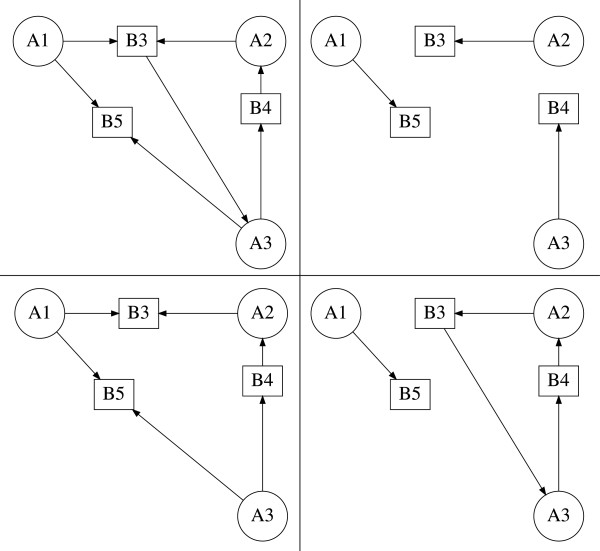
**Critical fragment and subgraphs of the reversible substrate inhibition mechanism.** Critical fragment S31,2,35,3,4 and constituent subgraphs of the reversible substrate inhibition mechanism computed by GraTeLPy. (top left) Critical fragment S3=1,2,35,3,4. (top right) Subgraph *g*_3_={[*A*_1_,*B*_5_],[*A*_2_,*B*_3_],[*A*_3_,*B*_4_]}. (bottom left) Subgraph g1=C3=A1,A3,A2B5,B4,B3. (bottom right) Subgraph g2={[A1,B5],C2=A2,A3B3,B4}.

A *subgraphg*={L_1_,L_2_,…,L_*s*_} of *G* consists of edges or cycles L_*i*_, *i*=1,…,*s*, where each species is the beginning of only one edge, or one path participating in a cycle. In other words, the edges and cycles in a subgraph are species mutually disjoint. The number of species nodes in a subgraph is defined as its *order*. The *subgraph weight* is defined using the product of the cycle weights (11) and the edges weights (10) of the cycles and edges in *g*

(12) $$K_{g}={\left(-1\right)}^{c}\underset{C\in g}{\prod }K_{C}\underset{E\in g}{\prod }(-K_{E}),$$

where *c* is the number of cycles in *g*. For example, the subgraph g={[A1,B5],C2=A2,A3B3,B4} with weight *K*_*g*_=−1 is shown in Figure 2 (bottom right).

Since more than one path can exist between species nodes via different reaction nodes in a bipartite digraph, the number of subgraphs through the same node sets may be greater than one. The set of all subgraphs *g* of order *k* with the same species nodes ${\bar{V}}_{1}=\{A_{i_{1}},\ldots A_{i_{k}}\}$ and reaction nodes ${\bar{V}}_{2}=\{B_{j_{1}},\ldots B_{j_{k}}\}$ sets is called a *fragment* of order *k* and is denoted by Ski1,…,ikj1,…,jk. For a fragment Ski1,…,ikj1,…,jk we define the number

(13) $$K_{S_{k}}=\underset{g\in S_{k}}{\sum }K_{g}$$

as the *fragment weight*. If $K_{S_{k}}<0$, then *S*_*k*_ is a *critical fragment*.

For example, the fragment S31,2,35,3,4 is shown in Figure 2 (top left) together with its three subgraphs g1=C3=A1,A3,A2B5,B4,B3, g2={[A1,B5],C2=A2,A3B3,B4} and *g*_3_={[ *A*_1_,*B*_5_],[ *A*_2_,*B*_3_],[ *A*_3_,*B*_4_]}. Each of the first two subgraphs *g*_1_ and *g*_2_ contains a positive cycle, and thus S31,2,35,3,4 is a critical fragment since

$$K_{S_{3}}=\underset{g\in S_{3}}{\sum }K_{g}=K_{g_{1}}+K_{g_{2}}+K_{g_{3}}=-1-1+1=-1<0.$$

In [3,22] it is shown that the coefficients of the characteristic polynomial (8) have the following graph-theoretic representation

(14) ak(u,w)=∑Ski1,…,ikj1,…,jkKSkwj1…wjkui1…uik,k=1,…,n.

Note that similar terms in *a*_*k*_ have been combined using summation over the subgraphs of a fragment (13) and (14) is in a simplified form. It follows by (14) that the correspondence between a fragment Ski1,…,ikj1,…,jk and a non-zero term in *a*_*k*_(*u*,*w*) is one-to-one. For example, the negative coefficient in *a*_3_(*u*,*w*) given in (9) corresponds to the critical fragment S31,2,35,3,4 shown in Figure 2 (top left).

Critical fragments corresponding uniquely to negative terms in (14) are important for the existence of instabilities as it is explained next.

### Instability criteria for the Jacobian and the bipartite digraph

Here we summarize classical results from bifurcation analysis [1] and more recent results relating graph-theoretic methods to instabilities [3,4,22].

*Multistability* often arises from a saddle-node bifurcation in an ordinary differential equations (ODE) model, [1,24]. If a saddle-node bifurcation occurs, then a real eigenvalue *λ*(*u*,*w*) of *J*(*u*,*w*) changes sign as the parameters (*u*,*w*) change values. Hence, a necessary condition for multistability arising from a saddle-node bifurcation is *a*_*n*_(*u*,*w*)= det(−*J*(*u*,*w*))=0 for some parameter values of (*u*,*w*) [1].

Often ODE models of biochemical mechanisms (4) have mass conservation relations reducing the rank *r* of the stoichiometric matrix *S* and the Jacobian *J*(*u*,*w*) to *r*<*n*, which means that the last non-zero coefficient in (8) is *a*_*r*_(*u*,*w*). Thus if a saddle-node bifurcation exists, then *a*_*r*_(*u*,*w*)=0 for some values of (*u*,*w*) [3]. Therefore a critical fragment Sri1,…,irj1,…,jr of order *r*, corresponding uniquely to a negative term in (14) for *k*=*r*, is required for a saddle-node bifurcation, and thus for multistability [3,22]. Thus the potential of a biochemical mechanism (1) for multistability depends on the structure of its bipartite digraph.

*Oscillations* in ODE models of biochemical mechanisms (1) often arise from Hopf bifurcation. It is shown in [3], that if a coefficient *a*_*k*_(*u*,*w*)≥0, *k*∈{1,…,*n*−1} is close to zero, then it is possible to choose parameter values for (*u*,*w*) such that oscillations arising from Hopf bifurcation occur.

The existence of a critical fragment Ski1,…,ikj1,…,jk of order *k*∈{1,…,*n*−1} makes it possible to minimize *a*_*k*_(*u*,*w*)≥0, *k*<*n* for some parameter values of (*u*,*w*) by increasing the magnitude of the corresponding negative term in *a*_*k*_(*u*,*w*). If there are mass conservation relations reducing the rank of the Jacobian matrix to *r*<*n*, a critical fragment Ski1,…,ikj1,…,jk of order *k*<*r* is required to detect possible oscillations in an ODE model (4) of a biochemical mechanism (1). Thus, the existence of oscillations in the ODE model of a biochemical mechanism (1) can also be determined by the structure of the bipartite digraph.

Patterns in a corresponding reaction–diffusion model to (4) usually arise as a result of *Turing instability*. Turing instability arises when a spatially homogeneous equilibrium is asymptotically stable in the absence of diffusion and becomes unstable when diffusion is added to the model [25]. For the existence of Turing instability, we study the matrix *J*(*u*,*w*)−*μD*, where *J*(*u*,*w*) is the Jacobian matrix (6), *D* is a diagonal matrix with positive diffusion coefficients *d*_*i*_>0, *i*=1,…,*n* on the diagonal and *μ*>0 is a parameter (*μ* represents an eigenvalue of the negative Laplacian) [25]. Turing instability is associated with a real eigenvalue of the matrix *J*(*u*,*w*)−*μD* passing through zero from left to right as parameter values are varied. In [4,22] it is shown that a necessary condition for Turing instability is the existence of a critical fragment Ski1,…,ikj1,…jk of order *k*<*n*. Thus, the potential of a biochemical mechanism to display Turing instability can be inferred from the structure of its bipartite digraph.

## Implementation

Recall that the existence of a critical fragment Sri1,…,irj1,…,jr in the bipartite digraph of a biochemical mechanism, where *r* is the rank of the stoichiometric matrix *S*, can induce multistability. Similarly a critical fragment Ski1,…,ikj1,…,jk of order *k*<*n* can induce Turing instability or even oscillations. On the other hand if no critical fragments of order *r* are found, then the existence of multistability can be excluded for any values of the parameters. Similarly if no critical fragments of order *k*<*n* are found, then the existence of Turing instability can also be excluded for any values of the parameters.

GraTeLPy enumerates all critical fragments of user-defined order k for a given biochemical mechanism, thus providing the user with information on the potential of a biochemical mechanism for multistability, oscillations or Turing instability.

**Figure 3:**
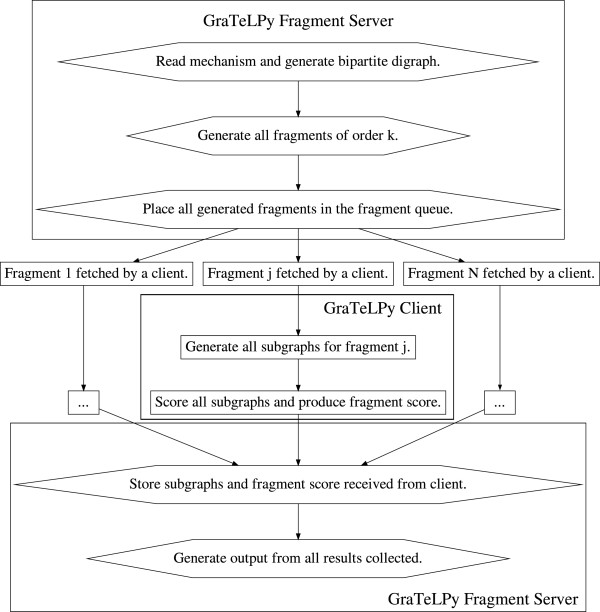
**Flowchart that summarizes the steps taken by GraTeLPy to find all critical fragments of a given order.** The division of tasks between a single server and one or more clients is highlighted. (top diamonds) The fragment server reads in the user-specified mechanism file and generates the bipartite digraph. The server generates all fragments of an user-defined order *k* and places them in a queue. (center rectangles) One or more client scripts fetch fragments off the queue and process them independently. For each fragment *S*_*k*_, a client generates all subgraphs and computes the weight of each subgraph. The subgraph weights are then added to compute the weight of the corresponding fragment. The client passes the computed data back to the server and fetches another fragment off the queue if the queue is not yet exhausted. (bottom diamonds) After preparing the fragment queue, the server waits for the results sent by the clients. Upon receipt of client-computed results for a fragment, the server stores these results if the fragment is found to have non-zero weight. Once the queue is exhausted, the server informs the user about the number of critical fragments discovered and generates other informative output.

We present in Figure 3 a flowchart that describes schematically the algorithm implemented by GraTeLPy.

First a biochemical mechanism is read from an user-provided input text file and its bipartite digraph is generated. Then all fragments Ski1,…,ikj1,…,jk of an user-defined order *k* are enumerated and placed in a computational queue. Each fragment from the queue will be further processed in order to compute its weight (top diamond nodes, Figure 3).

For each fragment Ski1,…,ikj1,…,jk in the queue, a linear sequence of operations is carried out (central rectangular nodes, Figure 3). First all subgraphs *g* of a fragment Ski1,…,ikj1,…,jk are enumerated, and the weight *K*_*g*_ of each subgraph *g* is computed. Then the weights of all subgraphs *g* contained in Ski1,…,ikj1,…,jk are added to compute the weight $K_{S_{k}}$ of the given fragment. At this point it is decided based on the sign of the weight $K_{S_{k}}$, if the fragment Ski1,…,ikj1,…,jk is critical, i.e., $K_{S_{k}}<0$ is satisfied.

Once all of the fragments from the queue have been processed, an output based on the potential of the biochemical mechanism for some desired instability is created (bottom diamond nodes, Figure 3). The information in the output includes the number of critical fragments of an user-defined order found by GraTeLPy. Based on the number of critical fragments found, GraTeLPy states if a biochemical mechanism meets the necessary condition for multistability or Turing instability, and if the mechanism can exhibit oscillations for some parameter values. In addition a list of all critical fragments of a given order detected by GraTeLPy can be provided.

Processing the queue of the enumerated fragments (central rectangular nodes, Figure 3) is inherently parallel as each fragment may be handled independently of all other fragments. To use this parallelism to our advantage, two scripts are created for GraTeLPy that implement a server role and a client role, respectively.

The server script takes care of actions in the top and bottom diamond nodes in Figure 3. The server creates the bipartite digraph, enumerates all fragments and places them in a queue (top diamond nodes). At the end the server collects all computed data from the client processes before displaying them for the user (bottom diamond nodes).

The client script deals with actions in the central rectangular nodes in Figure 3. The client fetches a fragment from the queue presented by the server, generates all subgraphs of the fragment, computes the weights of the subgraphs, computes the weight of the fragment, and reports all computed results back to the server. If the fragment queue has not been exhausted, then the client fetches another fragment and repeats these steps.

This server-client architecture allows the user to run one or multiple instances of the client script to analyze several fragments of a large mechanism in parallel. We discuss the technical details of the parallelization in more detail in Implementation challenges below.

In the following subsections we describe in detail the implementation of both fragment and subgraph enumeration as these parts presented considerable technical challenges during development.

### Fragment enumeration

It follows by the definition of a fragment given in Section The bipartite digraph of a biochemical mechanism that fragments are identified by the species and reaction indices of their subgraphs. A fragment Ski1,i2,…,ikj1,j2,…,jk of order *k* contains *k* unique species indexed by {*i*_1_,…,*i*_*k*_}, and *k* possibly repeated reactions indexed by {*j*_1_,…,*j*_*k*_}.

Suppose that a given biochemical mechanism has *N* species and *R* reactions. Using a combinatorial approach, we can generate all fragments of order *k* by pairing the Nk unique combinations of species with *R*^*k*^ combinations of reaction nodes. This approach generates Nk·Rk possible fragments that need to be filtered. This is because many of the combinatorially generated fragments do not exist in the bipartite digraph of a given biochemical mechanism.

To save computational time and cost we use a different approach. We note that each fragment Ski1,i2,…,ikj1,j2,…,jk contains one subgraph that consists of edges $\left[\;A_{i_{s}},B_{j_{s}}\right]$, *s*=1,…,*k*. Let us denote by |*E*_*i*_| (*i*=1,…,*n*_*i*_) the number of edges that a species *A*_*i*_ in a biochemical mechanism induces, or, is the starting node of. If we assume that each species *A*_*i*_ is on average the starting node of |*E*|=Avg(*E*_*i*_) edges, then this approach generates approximately Nk·|E|k fragments. Empirically, we observe that |*E*| is usually considerably less than some common values for the number of reactions *R*. Hence this latter approach generates fewer fragments than the former combinatorial approach. In fact, since fragments correspond uniquely to the subgraphs consisting of edges, using this method we generate only the fragments that are present in the bipartite digraph.

**Figure 4:**
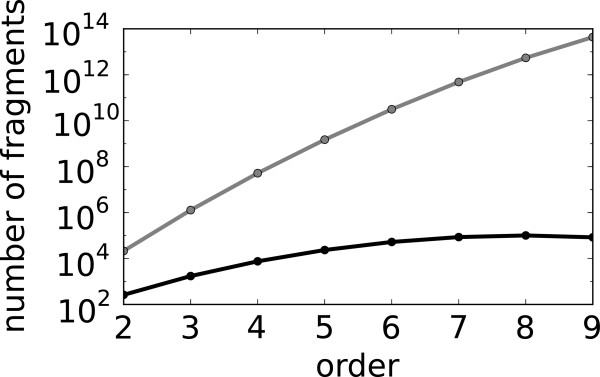
**Fragment enumeration for double-layer MAPK mechanism.** Number of fragments of different orders generated for the double-layer MAPK network (i) combinatorially (gray) and (ii) generated from the unique correspondence between fragments and edges-only subgraphs (black).

By using the method of one-to-one correspondence between fragments and subgraphs consisting of edges, we reduce the number of fragments generated by the combinatorial approach by multiple orders of magnitude. A reduction in the number of the generated fragments translates directly to a reduction in computational cost. Hence the latter approach for fragment generation is an important development in the implementation of GraTeLPy that allows for analyzing larger biochemical mechanisms. To highlight this reduction in computational cost we plot the number of fragments (of varying order) generated with both methods for the double-layer mitogen-activated protein kinase (MAPK) mechanism in Figure 4. The double-layer MAPK mechanism is discussed in more detail in the last example in Section Results and discussion.

### Subgraph enumeration

Given a fragment Ski1,i2,…,ikj1,j2,…,jk we generate all edges $\left[\;A_{i_{s}},B_{j_{s}}\right]$, positive paths $\left[\;A_{i_{s}},B_{j_{s}},A_{i_{l}}\right]$ and negative paths $\left[\;\bar{A_{i_{s}},B_{j_{s}},A_{i_{l}}}\right]$, where *l*,*s*=1,…,*k*, that are induced by the species and reactions of the fragment. We will refer collectively to edges, and positive and negative paths of a subgraph as *subgraph components*.

The subgraph components of a fragment Ski1,i2,…,ikj1,j2,…,jk are stored in a lookup table that lists for each species $A_{i_{s}}$ and corresponding reaction $B_{j_{s}}$ all subgraph components induced by the pair $\left(A_{i_{s}},B_{j_{s}}\right)$. The subgraph components of a fragment Ski1,i2,…,ikj1,j2,…,jk are generated as follows: 

(i) For a fragment Ski1,i2,…,ikj1,j2,…,jk each edge $\left[\;A_{i_{s}},B_{j_{s}}\right]$ (*s*=1,…,*k*) is identified and stored in the lookup table.

(ii) For each edge $\left[\;A_{i_{s}},B_{j_{s}}\right]$ in the lookup table, arcs starting at $B_{j_{s}}$, such as $\left(B_{j_{s}},A_{i_{l}}\right)$ (*l*=1,…,*k*) are identified. This way all positive paths induced by $\left(A_{i_{s}},B_{j_{s}}\right)$ are generated and added to the lookup table as part of the record for species $A_{i_{s}}$.

(iii) Similarly to (ii), for each edge $\left[\;A_{i_{s}},B_{j_{s}}\right]$ in the lookup table, arcs ending at $B_{j_{s}}$, such as $\left(A_{i_{l}},B_{j_{s}}\right)$ are identified. This way all negative paths induced by $\left(A_{i_{s}},B_{j_{s}}\right)$ are generated and added to the lookup table as part of the record for species $A_{i_{s}}$.

To gain some intuition on how subgraphs can be generated, we first describe a simple combinatorial approach before we introduce the method implemented by GraTeLPy. Suppose that for a fragment Ski1,i2,…,ikj1,j2,…,jk there are $\left\{L_{i_{1}},L_{i_{2}},\ldots ,L_{i_{k}}\right\}$ subgraph components induced by each species $\left\{A_{i_{1}},\ldots ,A_{i_{k}}\right\}$. We can generate combinatorially a subgraph *g* of Ski1,i2,…,ikj1,j2,…,jk by selecting at random one subgraph component per species since each species must be the starting node of exactly one component [3]. Using this approach we can generate combinatorially all possible combinations of subgraph components $\left|L_{i_{1}}|\cdot |L_{i_{2}}|\cdots |L_{i_{k}}\right|$, that represent all possible subgraph candidates of a fragment Ski1,i2,…,ikj1,j2,…,jk. For a fragment with |*L*|=Avg(*L*_*i*_) subgraph components per species on average, this method generates |*L*|^*k*^ possible subgraphs.

Even though this method guarantees that each species is the starting node of exactly one subgraph component, there may be combinations of paths that do not form cycles as defined in Sec. Mathematical background. This is because the end species node of a path has to be the starting species node of another path in a cycle [3]. If we use the combinatorial method for generating subgraphs, then all candidate subgraphs that do not satisfy the definition of a subgraph given in Sec. Mathematical background need to be removed which would increase the computational cost.

In the next two subsections we introduce the path graph and the cycle graph that will allow us to generate only the subgraphs that belong to a given fragment. The implementation of the algorithms associated with the path graph and the cycle graph by GraTeLPy will allow us to further reduce the computational cost.

#### Cycle detection: the path graph

We can avoid generating invalid subgraphs if paths are not joined combinatorially, but rather only paths that form cycles are joined. Recall that a cycle is a sequence of paths where the end species node of each path is the starting species node of exactly one other path in the sequence.

Next, we introduce *expanded paths*, where a negative path $\bar{\left[\;A_{i},B_{m},A_{j}\right]}$ is converted into two expanded paths [ A_*i*_,B_*m*_,A_*j*_] and [ A_*j*_,B_*m*_,A_*i*_] that are positive. This expansion is necessary as negative paths can be traversed in both directions as explained in Section The bipartite digraph of a biochemical mechanism. To enumerate all cycles of a given fragment Ski1,i2,…,ikj1,j2,…,jk, we construct the directed graph (digraph) *Φ*. The nodes of *Φ* correspond uniquely to the expanded negative paths and the positive paths of a fragment Ski1,i2,…,ikj1,j2,…,jk. We connect the nodes of the digraph *Φ* representing paths whose end nodes and starting nodes are the same. For example, there is a directed edge in *Φ* that starts at a node representing $\left[\;A_{i_{1}},B_{j_{1}},A_{i_{2}}\right]$ and ends at a node representing $\left[\;A_{i_{2}},B_{j_{2}},A_{i_{3}}\right]$. Self-loops in *Φ* from a node back to itself are also permitted and they correspond to paths of the form $\left[\;A_{i_{1}},B_{j_{1}},A_{i_{1}}\right]$.

To summarize, we generate a digraph *Φ* with the following properties: 

● The nodes *Φ*_*i*_ of *Φ* are the expanded negative paths and the positive paths of a given fragment Ski1,i2,…,ikj1,j2,…,jk.

● A directed edge (*Φ*_*i*_,*Φ*_*j*_) exists if and only if the end species node of the path corresponding to *Φ*_*i*_ is the starting species node of the path corresponding to *Φ*_*j*_. Self-loops (*Φ*_*i*_,*Φ*_*i*_) are permitted and correspond to positive paths of the form [ *A*_*i*_,*B*_*j*_,*A*_*i*_].

We refer to the digraph *Φ* as the *path graph*. For a fragment Ski1,i2,…,ikj1,j2,…,jk with a total of *P* paths that include all expanded negative paths and all positive paths of Ski1,i2,…,ikj1,j2,…,jk, the generation of the path graph *Φ* has time complexity O(P(P−1)).

To detect the cycles of the path graph *Φ*, and ultimately the cycles of a given fragment Ski1,i2,…,ikj1,j2,…,jk, an implementation of Johnson’s algorithm [26] provided by NetworkX [27] is used by GraTeLPy. For a fragment Ski1,i2,…,ikj1,j2,…,jk with a total number of *P* expanded negative paths and positive paths (corresponding uniquely to the nodes of *Φ*), *P*_*E*_ sequential relations between these paths (corresponding uniquely to the directed edges of *Φ*), and *P*_*C*_ cyclic sequential relations (corresponding uniquely to the cycles of *Φ*), the enumeration of all *P*_*C*_ cycles requires $O\left(\left(P+P_{E})(P_{C}+1\right)\right)$ units of time [26].

**Figure 5:**
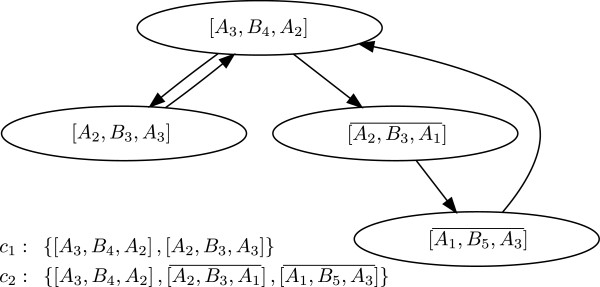
**Path graph for the critical fragment of the reversible substrate inhibition mechanism.** The path graph *Φ* and the detected cycles, that do not have repeated species as starting nodes of paths, of the critical fragment S3=1,2,35,3,4 shown in Figure 2. Only two cycles *c*_1_ and *c*_2_ that are reported previously in [3] are found.

Next we illustrate the construction and usage of the path graph *Φ* described above. The path graph *Φ* for the critical fragment S31,2,35,3,4 (see Figure 2) is shown in Figure 5, together with the cycles *c*_1_ and *c*_2_ produced by Johnson’s algorithm.

Some of the cycles of *Φ* enumerated by NetworkX correspond to closed paths with revisited nodes in the bipartite digraph *G*, and are therefore not cycles of *G*. This is the case because Johnson’s algorithm finds all cycles of all lengths of the path graph *Φ*. In our current implementation, we remove cycles of *Φ* that correspond to closed paths with revisited nodes of the bipartite digraph *G*. However, further optimization of Johnson’s algorithm is likely possible, so that only cycles that exist in the bipartite digraph *G* are generated in the first place.

#### Cycle combinations: the cycle graph

Suppose that *P*_*C*_ valid cycles of a given fragment Ski1,i2,…,ikj1,j2,…,jk have been found using the algorithm from the previous subsection. Possible candidates for subgraphs of Ski1,i2,…,ikj1,j2,…,jk can be constructed by creating all possible combinations of cycles. In total, there are ∑k=1PCPCk possible ways to combine *P*_*C*_ cycles into combinations of *k* cycles with no repeating cycles. Then, edges may have to be added to the combinations of cycles in order to construct the subgraphs of a fragment Ski1,i2,…,ikj1,j2,…,jk.

Generally, not all combinations of cycles or edges form subgraphs since such combinations may not contain every species of a fragment Ski1,i2,…,ikj1,j2,…,jk exactly once. Suppose that a given set of cycles has mutually disjoint species sets, but the orders of the cycles sum to less than *k*. In order to form a subgraph of a fragment Ski1,i2,…,ikj1,j2,…,jk we need to amend such a cycle combination with a set of edges whose species nodes are in Ski1,i2,…,ikj1,j2,…,jk, but not in any of the cycles. If on average a species *A*_*i*_ is the starting node of *E* edges and if on average we have to add *μ* edges to a cycle combination, then we generate combinatorially Eμ·∑k=1PCPCk possible subgraphs.

Many of the combinatorially generated cycle and edges combinations will have repeated species nodes, thus rendering such a combination of edges or cycles invalid as a subgraph. Hence we need to verify which of the generated combinations of cycles or edges are subgraphs of Ski1,i2,…,ikj1,j2,…,jk. If validating a subgraph requires O(1) units of time then, on average, validating all possible candidates for subgraphs has time complexity O(Eμ·∑k=1PCPCk)=O(PC!). In reality, validating a combination of cycles or edges as a subgraph has greater time complexity than O(1). Therefore, the computational cost will be greatly reduced if we can generate only subgraphs that require no further validation steps.

We use a similar approach to the one for finding the cycles of a given fragment. We will reduce the problem of generating cycle or edge combinations forming subgraphs to a problem that can be solved with available algorithms from the literature. To this end we generate an undirected graph *Γ* whose nodes correspond uniquely to the cycles of a given fragment Ski1,i2,…,ikj1,j2,…,jk, that are found using the path graph *Φ*. Drawing an edge between two nodes (representing cycles of Ski1,i2,…,ikj1,j2,…,jk) means that these two cycles do not share species nodes and can be combined as a part of a subgraph. Next, we formally define the undirected graph *Γ*

● A node *Γ*_*i*_ of *Γ* represents a cycle of a given fragment Ski1,i2,…,ikj1,j2,…,jk.

● An edge (*Γ*_*i*_,*Γ*_*j*_) exists if and only if the set of species nodes of the cycle represented by *Γ*_*i*_ and the set of species nodes of the cycle represented by *Γ*_*j*_ are disjoint.

We refer to the undirected graph *Γ* defined above as a *cycle graph*.

If a given set of cycles does not contain a number of species equal to the order of the subgraph constructed, then species-disjoint edges need to be added. To this end the problem of generating a subgraph of a given fragment Ski1,i2,…,ikj1,j2,…,jk can be reduced to finding all cliques in *Γ*. Recall that a *clique* is a set of nodes of an undirected graph such that every node is connected to every other node from the set [23]. To find all subgraphs of a fragment Ski1,i2,…,ikj1,j2,…,jk, its corresponding undirected graph *Γ* should be searched for all cliques. This is a standard problem in graph theory, known as clique enumeration, that can be solved using existing algorithms from the literature [28].

As an example, we construct the cycle graph *Γ* corresponding to the fragment S31,2,35,3,4 of the Reversible Inhibition reaction (2) shown in Figure 2 (top left). We use the fact that the cycles *c*_1_ and *c*_2_ of the path graph *Φ*, shown in Figure 5 have paths that share at least one species. Hence, the cycle graph *Γ* consists of two nodes corresponding to the two valid cycles *c*_1_ and *c*_2_ with no edge connecting them. Since the cycle graph *Γ* constructed from the valid cycles in Figure 5 is completely disconnected, we can choose one cycle at a time and attempt to construct a valid subgraph by adding edges to the cycle. If *c*_1_ is chosen, then the remaining nodes A_1_ and B_5_ form the edge [ A_1_, B_5_] yielding a valid subgraph of order 3, Figure 2 (bottom right). If *c*_2_ is chosen, then no other nodes remain. Thus the cycle *c*_2_ forms a valid subgraph of order 3, Figure 2 (bottom left).

When generating subgraphs of a fragment combinatorially, the number of subgraph candidates depends on the number of subgraph components of a given fragment. Using the improved algorithm (based on the path and cycle graphs) implemented by GraTeLPy, the number of generated subgraphs depends on the number of cycles in the path graph.

**Figure 6:**
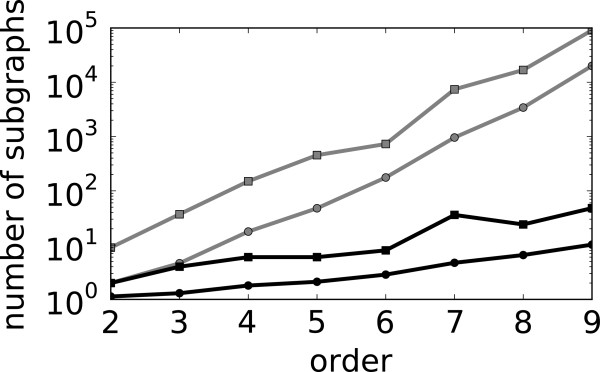
**Subgraph enumeration for double-layer MAPK mechanism.** Number of subgraph candidates generated for 100 randomly selected fragments (of indicated order) of the double-layer MAPK network. Gray: combinatorial approach, black: using the path and cycle graphs. Circles denote averages, squares denote maxima (maximal number of subgraph candidates generated for any one of 100 randomly selected fragments).

To compare the computational cost of the two approaches, we count the number of subgraphs generated in both cases for 100 randomly selected fragments of varying order for the double-layer MAPK network. The results are presented in Figure 6, and show that multiple orders of magnitude fewer subgraphs are generated by the path and cycle graph method in comparison to the combinatorial method.

### Implementation challenges

As an overarching principle, we have strived to reduce code duplication hence we reuse as many components as possible from open source libraries. To this end, we have used combinatorial standard libraries distributed with the programming language Python [29], NetworkX [27] for all graph-related operations, and matplotlib [30] for graphical output. We note however that matplotlib is an optional package and is not required for the core functionality of GraTeLPy.

Over the course of implementation of GraTeLPy we encountered combinatorial blowup and memory usage as major challenges. Thus we have designed GraTeLPy to minimize storage of fragments, subgraphs, and intermediate structures in memory. To this end we make considerable use of the standard Python library *itertools* and the concept of *generators* that allow us to transfer many results from one method to another with minimal memory footprint.

The cycle enumeration method provided by NetworkX [27] stores all detected cycles, causing memory shortage and overflow due to the large number of generated invalid cycles revisiting species. We have amended this library method to only store and return valid cycles of the bipartite digraph, i.e., those cycles that do not revisit species nodes.

Analyzing large networks is computationally unfeasible when only a single processor is used. Hence, we have parallelized the code. Python’s global interpreter lock causes threaded code to run slowly, so we have used the Multiprocessing module [29], which operates by using subprocesses rather than threads. We have implemented two parallelized versions of the code: 

1. Single multiprocessor machine. Here we use the Multiprocessing.Pool system, whereby the Multiprocessing module itself launches the requisite subprocesses. The code allows the user to specify the number of subprocesses that should be run (ideally this should match the number of processors available on the machine).

2. Multiple machines client/server. In this case, we launch a server process that generates the list of fragments to be analyzed. Clients can then be launched on any machine with a network connection to the server. These clients receive fragments from the server and pass back to the server the results of the analysis of the fragments. The server collates the responses.

Because the time spent analyzing a fragment is orders of magnitude higher than the time required to pass a representation of the fragment between a client and the server, the parallelization is extremely efficient. We have tested this client/server implementation with over 500 client processes, and the processing time scales very well.

## Results and discussion

GraTeLPy allows the user to enumerate critical fragments of an user-defined order. Thus biochemical mechanisms can be analyzed for their potential for some instability in an efficient way. The existence of multistability requires at least one critical fragment of order *r*, which is the rank of the stoichiometric matrix. If a critical fragment of order *k*<*n* exists, then oscillations may exist for some parameter values. The existence of Turing instability requires at least one critical fragment of order *k*<*n*, where *n* is the number of species.

Several examples of biochemical mechanisms of different sizes are presented in this section. We have used GraTeLPy to find the critical fragments of a given order in the bipartite digraph of each biochemical mechanism. The first three examples are smaller mechanisms and are used to verify the correctness of implementation of GraTeLPy, since their critical fragments have already been found elsewhere. Furthermore, we show that by using GraTeLPy, finding critical fragments in larger biochemical mechanisms such as the MAPK single-layer and MAPK double-layer networks becomes feasible. The median running time for finding the critical fragments for each biochemical mechanism is presented.

The models and data discussed in this section are available at https://github.com/gratelpy/gratelpy-supplementary-information.

### Reversible substrate inhibition

The reversible substrate inhibition model is analyzed for multistability in [3] using the graph-theoretic method presented here.

GraTeLPy reads in the biochemical mechanism from a plain text file.

Recall that the bipartite digraph of (15) shown in Figure 1.

The bipartite digraph of (15) contains one critical fragment S31,2,35,3,4 of order 3 found in [3]. GraTeLPy reproduces this fragment and its constituent subgraphs shown in Figure 2.

The median running time with one processor for finding the critical fragment S31,2,35,3,4 is 0.05 sec.

#### **Remark**

Note that the critical fragment S3=1,2,35,3,4 corresponds to the negative term in the last non-zero coefficient (9) of the characteristic polynomial of the Jacobian matrix (7). This suggests how we may choose parameter values for (*u*,*w*) so that a saddle-node bifurcation and multistability occur. The inequality *w*_4_>*w*_1_ should be satisfied, otherwise *a*_3_(*u*,*w*)>0. Also *u*_4_≫*u*_*i*_, *i*=1,2,3 so that *a*_3_(*u*,*w*) is close to zero. In general if Ski1,…,ikj1,…,jk is a critical fragment, then the species concentrations at equilibrium $u_{i_{s}}>0$, *s*=1,…,*k* should be chosen small and the rate functions $w_{j_{s}}>0$, *s*=1,…,*k* should be chosen large in order for a saddle-node bifurcation to occur.

As a future extension of GraTeLPy, we plan to make parameter choices for (*u*,*w*) such that some desired instability occurs available to the user.

### Glycolysis-Gluconeogenesis switch

Critical fragments of order smaller than *n*, the number of species in a biochemical mechanism, can induce Turing instability in a reaction–diffusion model [4] as well as oscillations in an ODE model [3].

The biochemical mechanism

(15) $$\begin{array}{llll} B_{1}: & \emptyset & \overset{k_{1}}{\underset{}{\to }} & A_{2},\\ B_{2}: & A_{2} & \overset{k_{2}}{\underset{}{\to }} & \emptyset ,\\ B_{3}: & A_{1}+A_{2} & \overset{k_{3}}{\underset{}{\to }} & A_{1}+A_{4},\\ B_{4}: & A_{4}+A_{6} & \overset{k_{4}}{\underset{}{\to }} & A_{2}+A_{1},\\ B_{5}: & A_{5}+A_{1} & \overset{k_{5}}{\underset{}{\to }} & A_{5}+A_{6},\\ B_{6}: & A_{5}+A_{4} & \overset{k_{6}}{\underset{}{\to }} & A_{3},\\ B_{7}: & A_{3} & \overset{k_{7}}{\underset{}{\to }} & A_{5}+A_{4},\\ B_{8}: & A_{5}+A_{2} & \overset{k_{8}}{\underset{}{\to }} & A_{7},\\ B_{9}: & A_{7} & \overset{k_{9}}{\underset{}{\to }} & A_{5}+A_{2},\\ B_{10}: & A_{4} & \overset{k_{10}}{\underset{}{\to }} & \emptyset , \end{array}$$

represents a glycolysis-gluconeogenesis switch and is studied for oscillations in [31]. It has been shown that the critical fragments (identified here by GraTeLPy as well) are the structural reason for the oscillations [31]. Based on the existence of the critical fragments, parameter values are chosen such that oscillations occur [31]. Similarly the mechanism (15) is studied for the existence of Turing instability in [4]. In fact, parameter values are found such that Turing instability exists in the reaction–diffusion model of (15).

**Figure 7:**
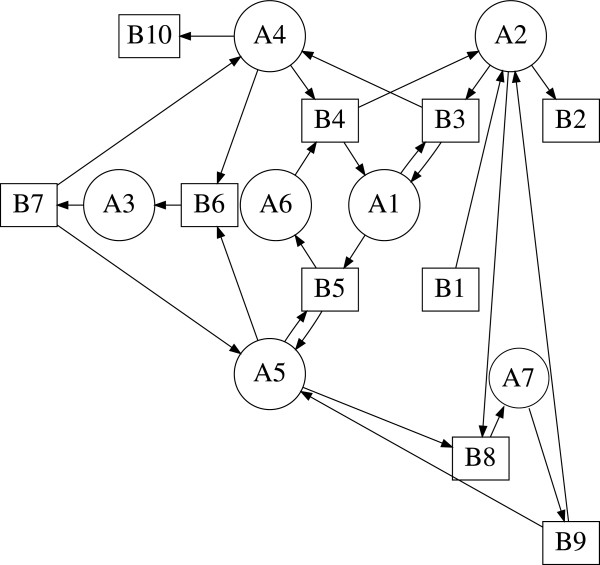
**Bipartite digraph of the Glycolysis-Gluconeogenesis switch mechanism.** Bipartite digraph of the glycolysisgluconeogenesis switch.

**Figure 8:**
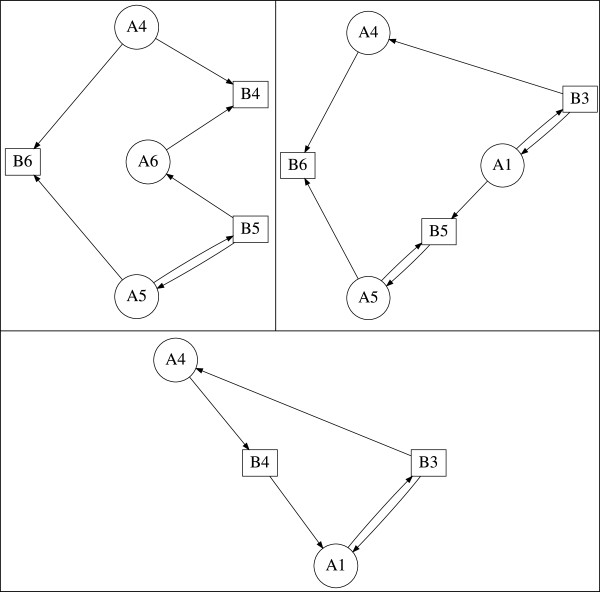
**Critical fragments of the Glycolysis-Gluconeogenesis switch mechanism.** Order 2 (bottom) and order 3 (top) critical fragments of the glycolysis-gluconeogenesis switch, reported in [4] and found by GraTeLPy.

The bipartite digraph of (15) is shown in Figure 7. The stoichiometric matrix associated with the biochemical mechanism (15) has rank 5. The biochemical mechanism meets the necessary criterion for Turing instability since critical fragments of order 1≤*k*≤5 exist [4]. In Figure 8 we show the critical fragments of order 2 and 3 reported in [4] and identified by GraTeLPy.

The median running time with one processor for finding the critical fragments of the bipartite digraph of the glycolysis-gluconeogenesis switch is 5.9 sec.

### Cdc42 network in yeast

A biochemical mechanism that describes the Cdc42 dynamics of yeast and cell polarity is studied in [32]. The corresponding reaction-diffusion model displays Turing instability and patten formation for some parameter values [32].

The biochemical mechanism of the Cdc42 network is given below

$$\begin{array}{rr} B_{1}: & E_{c}\overset{k_{1}}{\underset{}{\to }}E_{m},\\ B_{2}: & E_{m}\overset{k_{2}}{\underset{}{\to }}E_{c},\\ B_{3}: & \text{RT}+E_{m}\overset{k_{3}}{\underset{}{\to }}M,\\ B_{4}: & M\overset{k_{4}}{\underset{}{\to }}\text{RT}+E_{m},\\ B_{5}: & E_{m}+\text{RD}\overset{k_{5}}{\underset{}{\to }}E_{m},\\ vB_{6}: & M+\text{RD}\overset{k_{6}}{\underset{}{\to }}M+\text{RT},\\ B_{7}: & \text{RT}\overset{k_{7}}{\underset{}{\to }}\text{RD},\\ B_{8}: & E_{c}+\text{RT}\overset{k_{8}}{\underset{}{\to }}M,\\ B_{9}: & {\text{RDI}}_{m}\overset{k_{9}}{\underset{}{\to }}I+\text{RD},\\ B_{10}: & \text{RD}+I\overset{k_{10}}{\underset{}{\to }}{\text{RDI}}_{m},\\ B_{11}: & {\text{RDI}}_{c}\overset{k_{11}}{\underset{}{\to }}{\text{RDI}}_{m},\\ B_{12}: & {\text{RDI}}_{m}\overset{k_{12}}{\underset{}{\to }}{\text{RDI}}_{m}, \end{array}$$

where RD and RT denote the membrane-bound inactive and active form of Cdc42 respectively; I denotes cytoplasmic GDI that forms a membrane-bound complex with RD, RDI_m_, that detaches from the membrane and diffuses as RDI_c_ in the cytoplasm. The enzyme E is a complex that contains Cdc42-activating Cdc24 and exists in both a cytoplasmic and membrane-bound form, E_c_ and E_m_, respectively. If E is on the membrane, it can form a catalytic complex *M* together with RT, that aids activation of membrane-bound RD.

**Figure 9:**
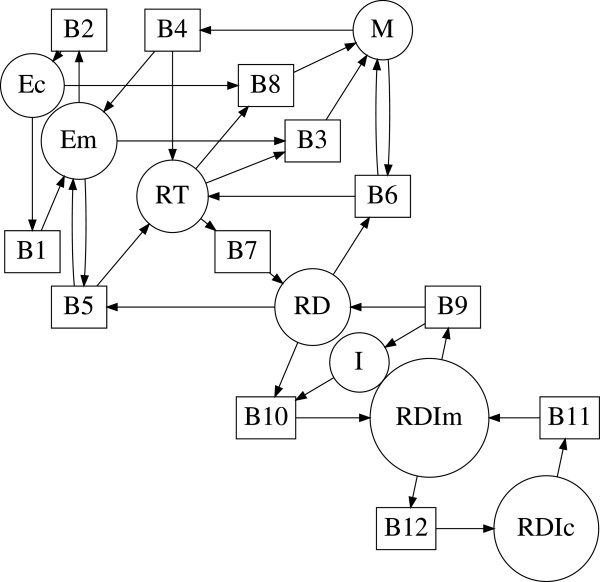
**Bipartite digraph of the yeast Cdc42 mechanism.** Bipartite digraph of the yeast Cdc42 network described in [32].

**Figure 10:**
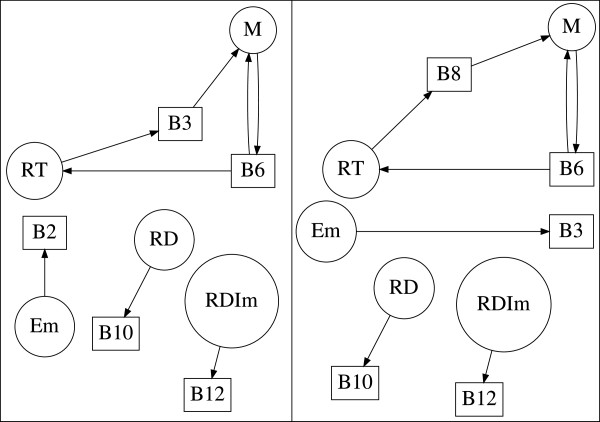
**Critical fragments of the yeast Cdc42 mechanism.** Critical fragments of order 5 of the yeast Cdc42 network reported in [32] and reproduced by GraTeLPy.

The bipartite digraph of the Cdc42 network is shown in Figure 9. The Cdc42 network has a corresponding stoichiometric matrix of rank 5. The necessary condition for Turing instability requires that a critical fragment Ski1,i2,…,ikj1,j2,…,jk of order 1≤*k*≤5 exists. GraTeLPy identifies 35 critical fragments – among which we find the two critical fragments reported in [32] and shown in Figure 10.

The median running time with one processor for finding the critical fragments of the bipartite digraph of the Cdc42 network is 9.7 sec, and with two processors 6.1 sec.

### Single-layer MAPK network

As the size of biochemical mechanisms increases, enumerating the critical fragments of their corresponding bipartite digraphs by hand becomes tedious and difficult. Using GraTeLPy we can find the critical fragments of a given order of larger mechanisms in a short period of time.

**Figure 11:**
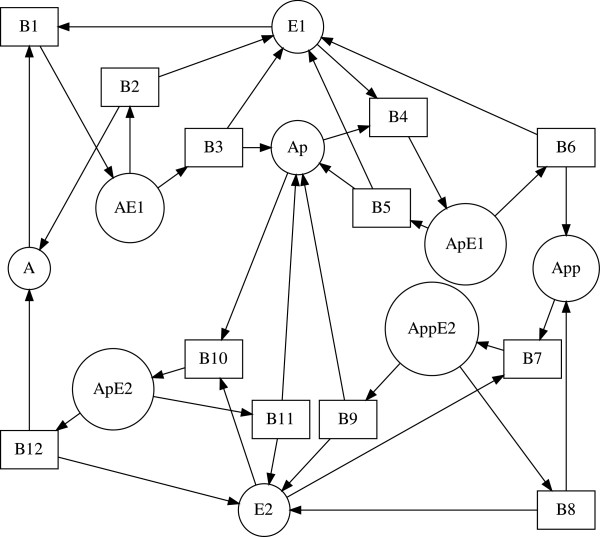
Bipartite digraph of the single-layer MAPK mechanism.

**Figure 12:**
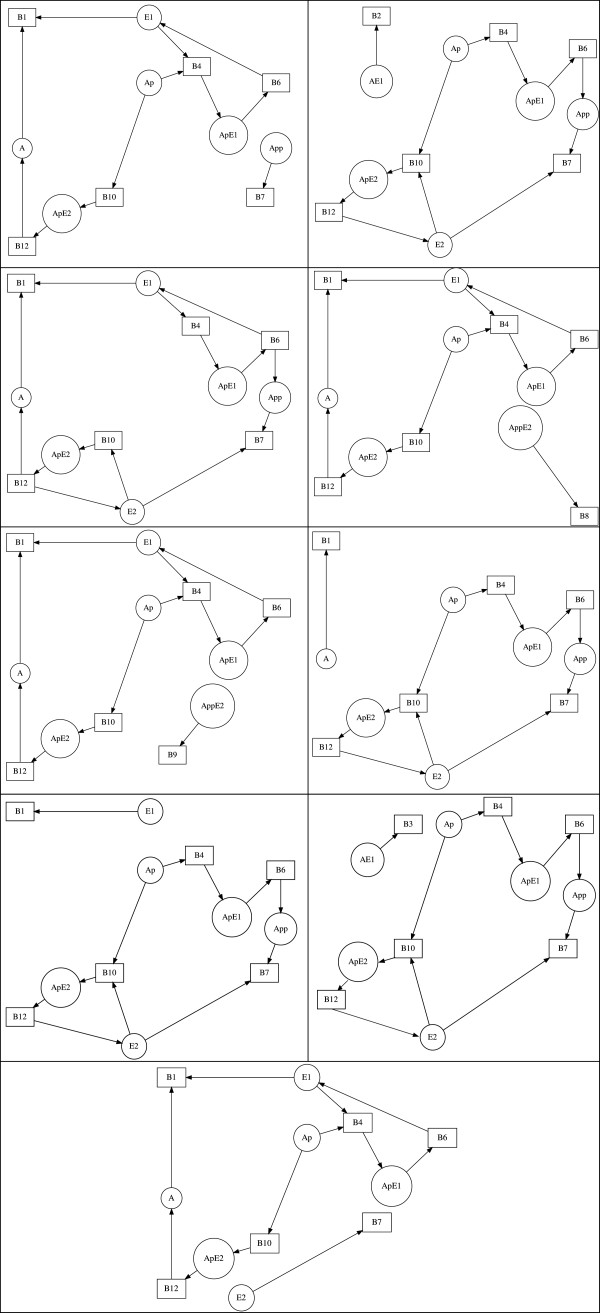
**Critical fragments of the single-layer MAPK mechanism.** Critical fragments of the single-layer MAPK network found by GraTeLPy.

An example of a larger biochemical mechanism that is difficult to analyze by hand is the single-layer MAPK network

$$\begin{array}{cccc} B_{1}: & A+E_{1} & \overset{k_{1}}{\underset{}{\to }} & {\text{AE}}_{1},\\ B_{2}: & {\text{AE}}_{1} & \overset{k_{2}}{\underset{}{\to }} & A+E_{1},\\ B_{3}: & {\text{AE}}_{1} & \overset{k_{3}}{\underset{}{\to }} & A_{p}+E_{1},\\ B_{4}: & A_{p}+E_{1} & \overset{k_{4}}{\underset{}{\to }} & A_{p}E_{1},\\ B_{5}: & A_{p}E_{1} & \overset{k_{5}}{\underset{}{\to }} & A_{p}+E_{1},\\ B_{6}: & A_{p}E_{1} & \overset{k_{6}}{\underset{}{\to }} & A_{\text{pp}},\\ B_{7}: & A_{\text{pp}}+E_{2} & \overset{k_{7}}{\underset{}{\to }} & A_{\text{pp}}E_{2},\\ B_{8}: & A_{\text{pp}}E_{2} & \overset{k_{8}}{\underset{}{\to }} & A_{\text{pp}}+E_{2},\\ B_{9}: & A_{\text{pp}}E_{2} & \overset{k_{9}}{\underset{}{\to }} & A_{p}+E_{2},\\ B_{10}: & A_{p}+E_{2} & \overset{k_{10}}{\underset{}{\to }} & A_{p}E_{2},\\ B_{11}: & A_{p}E_{2} & \overset{k_{11}}{\underset{}{\to }} & A_{p}+E_{2},\\ B_{12}: & A_{p}E_{2} & \overset{k_{12}}{\underset{}{\to }} & A+E_{2}, \end{array}$$

whose bipartite digraph is shown in Figure 11. The MAPK network is a well-known example of a multistable system [5,33]. The necessary condition for multstability requires the existence of a critical fragment of order equal to the rank of the stoichiometric matrix. Since the rank of the stoichiometric matrix for the MAPK network equals 6, using GraTeLPy, we enumerate all critical fragments of order 6. The 9 critical fragments of order 6 of the MAPK network are shown in Figure 12.

The median running time with two processors for finding the critical fragments of the bipartite digraph of the single-layer MAPK network is 10.7 sec.

### Double-layer MAPK network

For large biochemical mechanisms the number of critical fragments of a given order may grow into the dozens or hundreds. Thus the task of enumeration by hand of all critical fragments of a given order becomes unfeasible, but can be accomplished with the help of GraTeLPy.

An example of a larger biochemical mechanism is provided by the double-layer MAPK network which has 12 species and 18 reactions

(16) $$\begin{array}{cccc} B_{1}: & A+E_{1} & \overset{k_{1}}{\underset{}{\to }} & {\text{AE}}_{1},\\ B_{2}: & {\text{AE}}_{1} & \overset{k_{2}}{\underset{}{\to }} & A+E_{1},\\ B_{3}: & {\text{AE}}_{1} & \overset{k_{3}}{\underset{}{\to }} & A_{p}+E_{1},\\ B_{4}: & A_{p}+E_{1} & \overset{k_{4}}{\underset{}{\to }} & A_{p}E_{1},\\ B_{5}: & A_{p}E_{1} & \overset{k_{5}}{\underset{}{\to }} & A_{p}+E_{1},\\ B_{6}: & A_{p}E_{1} & \overset{k_{6}}{\underset{}{\to }} & A_{\text{pp}}+E_{1},\\ B_{7}: & A_{\text{pp}}+E_{1} & \overset{k_{7}}{\underset{}{\to }} & A_{\text{pp}}E_{1},\\ B_{8}: & A_{\text{pp}}E_{1} & \overset{k_{8}}{\underset{}{\to }} & A_{\text{pp}}+E_{1},\\ B_{9}: & A_{\text{pp}}E_{1} & \overset{k_{9}}{\underset{}{\to }} & A_{\text{ppp}}+E_{1},\\ B_{10}: & A_{\text{ppp}}+E_{2} & \overset{k_{10}}{\underset{}{\to }} & A_{\text{ppp}}E_{2},\\ B_{11}: & A_{\text{ppp}}E_{2} & \overset{k_{11}}{\underset{}{\to }} & A_{\text{ppp}}+E_{2},\\ B_{12}: & A_{\text{ppp}}E_{2} & \overset{k_{12}}{\underset{}{\to }} & A_{\text{pp}}+E_{2},\\ B_{13}: & A_{\text{pp}}+E_{2} & \overset{k_{13}}{\underset{}{\to }} & A_{\text{pp}}E_{2},\\ B_{14}: & A_{\text{pp}}E_{2} & \overset{k_{14}}{\underset{}{\to }} & A_{\text{pp}}+E_{2},\\ B_{15}: & A_{\text{pp}}E_{2} & \overset{k_{15}}{\underset{}{\to }} & A_{p}+E_{2},\\ B_{16}: & A_{p}+E_{2} & \overset{k_{16}}{\underset{}{\to }} & A_{p}E_{2},\\ B_{17}: & A_{p}E_{2} & \overset{k_{17}}{\underset{}{\to }} & A_{p}+E_{2},\\ B_{18}: & A_{p}E_{2} & \overset{k_{18}}{\underset{}{\to }} & A+E_{2}. \end{array}$$

Similarly to the single-layer MAPK network, the double-layer MAPK network is known to display multistability. The stoichiometric matrix for the double-layer MAPK network has rank 9. Therefore the necessary condition for multistability requires the existence of at least one critical fragment of order 9. GraTeLPy detects 88 critical fragments of order 9. The list of all critical fragments of order 9 of the bipartite digraph of (17) can be obtained from https://github.com/gratelpy/gratelpy-supplementary-information.

The median running time of each client for finding the critical fragments of the bipartite digraph of the double-layer MAPK network is as follows: 141 sec with 100 clients, 270 sec with 50 clients and roughly 4 hours with a single client.

## Conclusions

We have implemented a graph-theoretic method that allows for parameter-free model testing of biochemical mechanisms with mass action kinetics for multistability, oscillations and Turing instability. GraTeLPy is open-source and is based on a free software. GraTeLPy enables users to identify the graph structures referred to as critical fragments that can be responsible for the existence of some instability in a differential equations model of a biochemical mechanism (1).

At present, GraTeLPy expects that the user converts a biochemical mechanism to a text format such as the one presented in (15). In a future release we plan to include additional functionality so that biochemical mechanisms provided in SBML [34] and other formats can be analyzed. A list of the critical fragments of a user-defined order is provided upon completion.

We plan a future extension of GraTeLPy where choices of parameter values such that some desired instability occurs will be offered to the user. This extension will be based on the existence of a critical fragment and its one-to-one correspondence with a negative term in a coefficient of the characteristic polynomial (See Remark in the Reversible substrate inhibition Example).

We also plan to combine GraTeLPy with a new analytic method, local perturbation analysis (LPA) [35,36], in order to test biochemical mechanisms for pattern formation.

An extension of GraTeLPy to multigraphs [37] that can be used for the analysis of gene regulatory networks [38] is also left as a future extension.

## Availability and requirements

GraTeLPy is available from https://github.com/gratelpy/gratelpy and has the following requirements: Python 2.6 or 2.7 and NetworkX 1.6 or above.

## Competing interests

The authors declare that they have no competing interests.

## Authors’ contributions

GRW and MH implemented the software. MM provided the mathematical background. GRW, MH and MM wrote the manuscript. All authors read and approved the final manuscript.

## Supplementary Material

Additional file 1 GraTeLPy Manual: A Practical Software Guide. Click here for file
