# Supplementary material for: GraTeLPy: graph-theoretic linear stability analysis
Source: BMC Syst Biol. 2014 Feb 27;8:22. doi: 10.1186/1752-0509-8-22 (PMC4016541; doi:10.1186/1752-0509-8-22)
Supplement: Additional file 1 — GraTeLPy Manual: A Practical Software Guide. [file 1752-0509-8-22-S1.pdf]

GraTeLPy Manual: A Practical Software Guide

Georg R Walther*[1], Matthew Hartley[1] and Maya Mincheva[2]

[1]Computational and Systems Biology, John Innes Centre, Norwich Research Park, Norwich, UK,
[2]Department of Mathematical Sciences, Northern Illinois University, DeKalb, IL, 60115, USA

Email: Georg R Walther*- georg.walther@jic.ac.uk - gratelpy@gmail.com;

*Corresponding author

Introduction

In this guide GraTeLPy refers to GraTeLPy Version 0.2.

Here we describe how to download, install, and use GraTeLPy. To continue improving our software, we welcome reports of any issues that arise with GraTeLPy under the following address: https://github.com/gratelpy/gratelpy/issues.

In addition to this guide, more documentation is available from https://github.com/gratelpy/gratelpy.

Download

GraTeLPy is a Python Package

GraTeLPy is primarily a Python package: the code for GraTeLPy is available openly and can be incorporated by programmers familiar with Python into their own code.

The main purpose of GraTeLPy is to enumerate all critical fragments of a given order in the bipartite digraph of a biochemical mechanism. We provide a set of Python scripts that perform the standard sequence of steps necessary for this purpose, Figure 3. These scripts are easy to use for the Python novice and do not require knowledge of programming in Python – they do however require the user to install GraTeLPy as a Python package. This also requires that all Python packages that GraTeLPy depends on are available on the system of the user.

Upon installation of GraTeLPy, these scripts are invoked directly from the terminal on both Linux and Mac OSX or the console on Windows.

Python is a programming language that is available free of charge for all major operating systems such as Windows, Mac OS, and Linux. Further, all Python packages that GraTeLPy depends on are also available

free of charge.

GraTeLPy has the following requirements: Python 2.6 or 2.7 and NetworkX 1.6 or above.

GraTeLPy as Python Package on Windows

We have tested GraTeLPy with Windows 7 and recommend the following steps to install GraTeLPy as a Python package on your Windows machine.

As Windows does not come with Python by default we strongly recommend installing a recent version of available Python distributions that are available free of charge. We have tested successfully GraTeLPy with recent versions of both Continuum Analytics Anaconda (http://continuum.io/downloads) and Enthought Canopy Express (https://www.enthought.com/canopy-express/).

After installing either one of these distributions it is advisable to make certain that Python is installed correctly. This is straightforward in the command line tool. On Windows 7, to open the command line tool click on *Start*, *All Programs*, *Accessories*, *Command Prompt*. Alternatively, hold down the *Windows key* and press the *R* key. Type *cmd* into the appearing window and confirm. In the command line tool, run *python* and confirm that you can either see "Enthought Canopy Python 2.7.3" (or similar) or "Python 2.7.5 Anaconda 1.8.0" (or similar) – depending on the Python distribution you installed.

Download the source code of GraTeLPy from https://pypi.python.org/pypi/GraTeLPy and unpack the source code to a convenient location on your hard disk.

In the command line tool, navigate to the location where you unpacked the source code of GraTeLPy. Make certain that you can see the file *setup.py* on your current path by typing *dir* followed by *Enter*.

To install GraTeLPy, type *python setup.py install*. This should download and install any Python packages you may miss and install GraTeLPy.

GraTeLPy as Python Package on Mac OS and Linux

Mac OS and Linux usually provide Python by default. Older versions of these operating systems may provide outdated Python below version 2.6. To identify your Python version, open a command window (terminal) and run *python*. Unfortunately, we are unable to offer GraTeLPy on systems with Python below version 2.6 since NetworkX requires at least version 2.6 of Python.

The easiest way to install GraTeLPy on Mac OSX and Linux is through a command line program called *pip*. This is a software package that downloads Python packages from a central repository, *PyPI*, and installs the desired package together with all dependencies. If on the command line you cannot run *pip*,

there are multiple options you have available to install *pip*:

- On the command line (Mac OSX and Linux) run "sudo easy_install pip",
- install the Linux package "python-pip".

More information and a variety of other options can be found here:
http://www.pip-installer.org/en/latest/index.html.
Further, it is possible to install GraTeLPy without *pip*. To do so, download the source code of GraTeLPy from https://pypi.python.org/pypi/GraTeLPy and unpack the source code into an accessible location. In your terminal, navigate to the location of the GraTeLPy source code and make certain that you can see *setup.py* on your current path. To install GraTeLPy run the following command: *sudo python setup.py install*.
If you are unable to install GraTeLPy because you do not have NetworkX installed repeat the above steps for NetworkX first and then install GraTeLPy. Specifically, download the NetworkX source code from https://pypi.python.org/pypi/networkx/ (for the source code, choose the file called "networkx-1.8.1.tar.gz" or "networkx-1.8.1.zip – the version number may vary) and unpack the source code to an accessible location. On the command line, navigate to the location where you unpacked the NetworkX source code, make certain you can see "setup.py", and invoke "sudo python setup.py install". Once installation of NetworkX is complete, repeat these steps with GraTeLPy.

Use

Here we first discuss the mechanism format expected by GraTeLPy. Further below, we discuss how to invoke GraTeLPy.

Mechanism Format

To analyze a model, we need to convert it to the reaction mechanism format expected by GraTeLPy. The main text of this manuscripts gives an example of what the format looks like, Equation (15). Furthermore, GraTeLPy supplies a number of mechanisms. If you download and unpack the GraTeLPy source code (as described above) you will find these mechanisms in directory "gratelpy/mechanisms" inside the GraTeLPy source code directory. You can also view all mechanisms at this location:
https://github.com/gratelpy/gratelpy/tree/master/gratelpy/mechanisms.

The GraTeLPy mechanism format is human-readable and very close to the way we usually write reaction mechanisms with only a few exceptions:

- Chemical species are written in square brackets "[]",
- the end of each elemental reaction is denoted by a semicolon,
- and the rate constant is written following this semicolon.

This format also allows us to comment individual lines of mechanism files by typing a hash key, "#", at the beginning of the line. Using comment lines in our mechanism file, we can document useful information such as the name of the mechanism and the number of chemical species, Equation (15).

At present we need to convert our mechanism description to this expected format for use with GraTeLPy. However, we plan to implement conversion functionality so that GraTeLPy can also read mechanisms in standard formats, such as SBML [**?**].

Using the GraTeLPy Scripts

All GraTeLPy scripts can be evoked from the command window from all supported operating systems: Windows, Mac OS, and Linux.

Once GraTeLPy is installed you may test your installation by invoking scripts "gratelpy_test" and "gratelpy_time". The former script runs a series of tests that ensure that the GraTeLPy works correctly algorithmically while the latter script runs a series of analyses that show how fast GraTeLPy runs on your system.

To analyze your own mechanism you need to use scripts *gratelpy_fragment_server* and *gratelpy_subclient*. To see a reminder of their intended use in your terminal, call either one of these scripts without arguments. The server expects that you indicate the name of your mechanism file and the number of species in your mechanism. Optionally, you may indicate the fragment order you wish to test for as a third argument – if you do not provide an order, GraTeLPy will test for critical fragments of order equal to the rank of the stoichiometric matrix.

Suppose you save your mechanism, that contains four species, in *mechanism.txt*, and the stoichiometric matrix of your mechanism has order three. Then to test your mechanism for critical fragments of order three, you may either run *gratelpy_fragment_server mechanism.txt* or *gratelpy_fragment_server mechanism.txt 4 3*. To test your mechanism for critical fragments of order two you can run *gratelpy_fragment_server mechanism.txt 4 2*.

The fragment server presents the user with a number of fragments it generated, and other informative output, once it is ready to serve fragments to client processes. The client script requires the same arguments as the server script except for the order of the fragments you are interested in. For the above example, you can begin analyzing fragments provided by the server by starting a client process in another terminal window: *gratelpy_subclient mechanism.txt 4*. Notice that you can start multiple clients by repeating these steps in order to analyze your mechanism in parallel.

If you wish to run your server process and your client process on separate computers, you need to provide the *hostname* of the computer that runs the server: *gratelpy_subclient mechanism.txt 4 hostname*. We have tested this successfully on Linux clusters.
